# Novel Oronasal Drainage for Long COVID: Proposed Mechanisms—Case Report

**DOI:** 10.3390/v17020210

**Published:** 2025-01-31

**Authors:** Claudia Lorenz, Roland Frankenberger

**Affiliations:** 1 Department of Operative Dentistry, Sanitätszentrum Pfreimd, Schloßbergstr. 1, 92536 Pfreimd, Germany; claudia-lorenz@posteo.de; 2 Department of Operative Dentistry and Endodontics, Dental School, University of Marburg and University Medical Center Giessen and Marburg, 35039 Marburg, Germany

**Keywords:** Long COVID, oronasal drainage (OND), myalgic encephalomyelitis/chronic fatigue syndrome (ME/CFS), ostia, oral, nose, case report

## Abstract

Long COVID, potentially emerging post COVID-19 infection, involves extreme health challenges. Based on current literature in the field, we propose a novel approach to Long COVID treatment based on epipharyngeal abrasive therapy targeting ostia of the oral and nasal mucosa, having been identified for the first time. The presented case report documents the application of innovative oronasal drainage (OND), a novel treatment integrating physiological, biochemical, and fluid mechanical components simultaneously. OND led to remarkable improvements and even remissions of various symptoms, along with enhanced hand blood circulation. While the case suggests potential efficacy in Long COVID therapy, acknowledging inherent limitations is essential and its impact needs further validation through clinical trials.

## 1. Introduction

The oral and pharyngeal mucosa are fundamental targets of severe acute respiratory syndrome coronavirus 2 (SARS-CoV-2) [1,2]. A recent meta-analysis indicated a 45% risk of persistent symptoms four months post-infection [3], with a higher incidence among females [4]. Cumulative reinfections further elevate the risk of Long COVID [5].

The prevalence of chronic epipharyngitis in Long COVID reaches 100% [6]. Applying a cotton laryngeal swab, inserted in 0.5% zinc chloride (ZnCl_2_) solution, to the epipharynx has shown significant improvements in chronic cough [7]. Notably, epipharyngeal abrasive therapy (EAT), involving the abrasive removal of mucus with 0.5% ZnCl_2_ solution, has received approval for treating acute COVID infections and Long COVID in Japan [7,8,9], supported by a recent comprehensive study [6].

In Germany, no therapies for the treatment of Long COVID are currently approved, besides strong evidence that SARS-CoV-2 can persist [10,11,12,13,14,15]. In this context, we introduce an innovative therapeutic approach—the oronasal drainage (OND), invented by Claudia Lorenz—targeting the lymphoepithelial tissue of the oral and nasal mucosa (ONM) using 0.5% ZnCl_2_ solution. This report marks the first global description and systematic use of the OND method, developed and implemented in Germany. The primary objective of OND is the direct elimination of infected cells at the point of entry. In contrast to EAT, OND abstains from the abrasive removal of mucus. Instead, it employs a spiral excretion technique that follows the natural link of secretion. OND offers significant advantages over EAT, as it minimizes or entirely eliminates bleeding and spares the patient from experiencing pain [6]. Furthermore, OND targets multiple sites with the objective of more effectively reducing the possible presence of actively replicating virus, consequently leading to a greater alleviation of manifestations [15,16].

This treatment was administered to a 57-year-old woman experiencing Long COVID and Myalgic encephalomyelitis/chronic fatigue syndrome (ME/CFS), involving multiple OND per day over five consecutive days in September 2023 and no evidence of SARS-CoV-2 infection at that time. Remarkably, the outcomes of the OND intervention demonstrated systemic, pulmonary, cardiovascular, gastrointestinal, musculoskeletal, and neuropsychiatric improvements. Additionally, remissions of gynecological and gastrointestinal complaints were observed, accompanied by enhanced palmar blood circulation. Enhancement enabled patient’s partial resumption of duties including public appearances.

## 2. Materials and Methods

### 2.1. Safety and Ethics

The OND method is currently in the experimental phase, prioritizing stringent adherence to safety and ethical standards through meticulous clarification, documentation, and responsible handling.

Safety measures are implemented, treating removed secretions as potentially infectious. Risk of infection through aerosol-generating procedures is mitigated by employing a HEPA air purifier during OND [17]. The practitioner wears personal protective equipment, including goggles, FFP2 mask, visor, hooded suit, disposable gloves, and shoe covers. Additionally, the individual undergoing OND wears protective goggles with cotton pads for further protection.

### 2.2. Materials

Essential materials for OND include sterile 15 cm long, straight cotton swabs soaked in 0.5% ZnCl_2_ solution and lidocaine pump spray. The 0.5% ZnCl_2_ solution, a standard component for EAT in Japan [6,7,8,9], was used off-label for a single individual in Germany for OND, ensuring responsible product handling. A case report documented a patient’s self-administration of EAT, demonstrating improved renal function and relief from a sore throat, underscoring the low risk in self-treatment [18]. The OND employs sterile straight cotton swabs, enabling targeting of ostia and crypts and rotation, unlike the EAT that utilizes angled instruments on a flat surface.

Topical pharyngeal anesthesia, administered using a lidocaine pump spray in a supine position, effectively minimizes the possibility of a gagging reflex during OND [19].

### 2.3. Patient Selection Criteria

Patient selection involves confirmed SARS-CoV-2 infection through PCR testing and a diagnosis of Long COVID, possibly caused by virus persistence [10,11,12,13,14,15]. Exclusion criteria encompass minors and probands already participating in studies elsewhere, ensuring a targeted focus on the unique aspects of OND in the context of treatment in this population.

### 2.4. Overview of OND

Despite the potential severity of symptoms associated with Long COVID, there are currently only off-label treatments available in Germany [20]. Introduced for the first time, OND is an evolution of EAT from Japan, involving the abrasive removal of mucus from the epipharynx using a 0.5% ZnCl_2_ solution [6,7,8,9] OND represents an alternative therapeutic approach for Long COVID, integrating simultaneously physiological, biochemical, and fluid mechanical components to expel exocrine secretions and initiate a healing cycle.

#### 2.4.1. Site of Action

**Figure 1:**
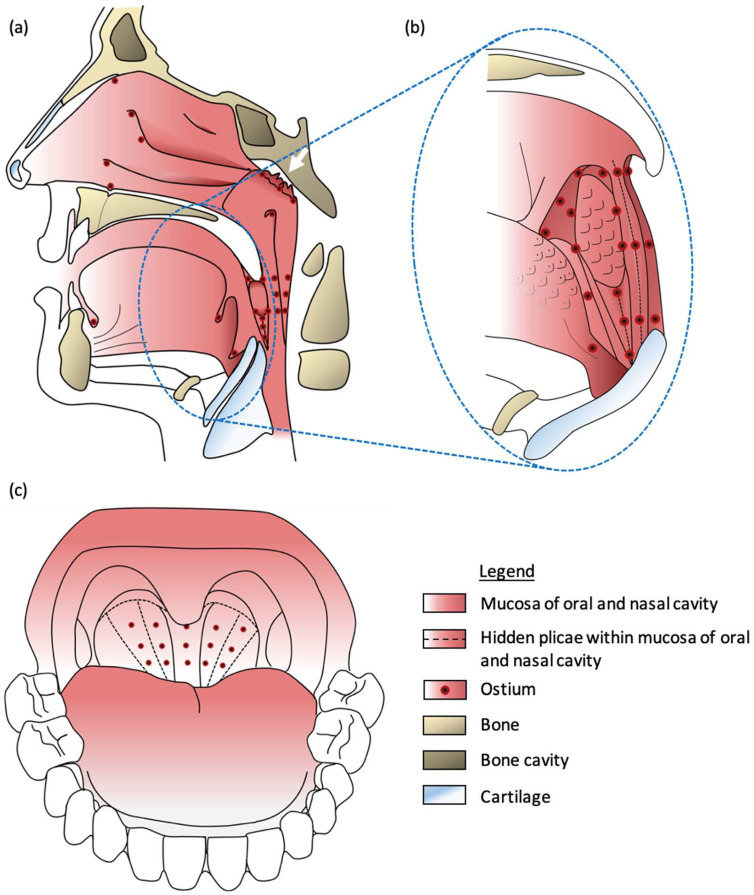
Proposed site of action of Oronasal Drainage (OND) for Long COVID treatment. (**a**) Schematic depiction of the human oral and nasal cavity, where the white arrow signifies the region for epipharyngeal abrasive therapy, and the red-black dots indicate ostia as the site of action for OND. (**b**) Schematic representation of the human oral cavity highlights numerous ostia near the tonsilla palatina, with hidden plicae unfolding through mechanical stimulation using cotton swabs. The illustration shows an enlargement of (**a**), highlighted with a blue dashed outline. (**c**) Schematic representation of the human oral cavity illustrates the arrangement of ostia within the oropharynx. Our hypothesis posits that the opening of obstructed ostia is facilitated by biochemical stimulation using 0.5% zinc chloride (ZnCl_2_) solution.

OND precisely targets the lymphoepithelial tissue in the ONM, where the epithelium houses angiotensin-converting enzyme 2, facilitating the entry of SARS-CoV-2 into host cells [2]. Specifically, the focus is on the less-explored and first time identified ostia, partly hidden inside crypts and plicae, of mucosa-associated lymphoid tissue (MALT), including tonsilla lingualis, tonsilla palatina, tonsilla pharyngealis, and tonsilla tubaria [21,22] (Figure 1a–c). Given the established transmission routes of SARS-CoV-2 through the eye, nose, or mouth [2,23,24], and in consideration of viral persistence on the tongue [10], adenoids and tonsils [25], we hypothesize that the entry points primarily coincide with the exit points of the virus [15].

#### 2.4.2. Physiological Component

Recent research supports the exploration of ostia, especially in the ONM, indicating their sphincter function [26]. Mechanical stimulation, such as with cotton swabs, exposes ostia within mucosal plicae and crypts.

#### 2.4.3. Biochemical Component

Cystic fibrosis transmembrane conductance regulator and SARS-CoV-2 accessory protein ORF3a form the biochemical basis for OND, considering their roles in chloride ions transport [27] and altered dynamics of chloride channels [28], respectively.

The 0.5% ZnCl_2_ solution demonstrates antibacterial properties within the oral cavity [29], and its anti-inflammatory effect in the epipharynx is evidenced by a suppressed expression of TNF-α, IL-6, and decreased number of CD4(+) T cells alongside symptomatic recovery [8,30]. We therefore hypothesize that the opening of blocked ostia is facilitated by the chloride ions being present in the ZnCl_2_ solution [28].

#### 2.4.4. Fluid Mechanical Component

OND is proposed to rely on a mechanism involving clockwise rotation, possibly linked to cell differentiation [31] and the structure of the SARS-CoV-2 virus [28,32]. This technique, executed in a spiral, clockwise manner from the patient’s perspective using extended cotton swabs, facilitates the absorption of secretions due to mucus adhesion [9]. The absorbed secretion onto the cotton swab forms a long thread, resembling a spindle [33].

#### 2.4.5. Secretions

OND aims to expel inflammatory fluids, potentially containing SARS-CoV-2 parts [16], through tears, nasal secretions, and saliva along the most direct route. Fluid management involves wiping tears, gently sniffing nasal secretions into a cellulose cloth, and expectorating saliva into a designated vessel. Large respiratory droplets carrying SARS-CoV-2 virus deposit due to gravitational force [34]. Utilizing a pillow to enhance the natural flow of these fluids, the individual consistently assumes a slightly forward-leaning position, typically seated with the head bent forward.

#### 2.4.6. Side Effects

Immune responses induced by OND, resembling a flu-like experience, parallel the regression of an acute viral infection [35]. Thus, essential parameters, including oxygen saturation, heart rate, and blood pressure, are assessed before and after each treatment day. Due to the possibility of aspirating potentially infectious saliva secretions during the spitting process [2], there is a risk of herpes simplex virus 1 (HSV-1) reactivation [36].

#### 2.4.7. Treatment Cycle

**Figure A1:**
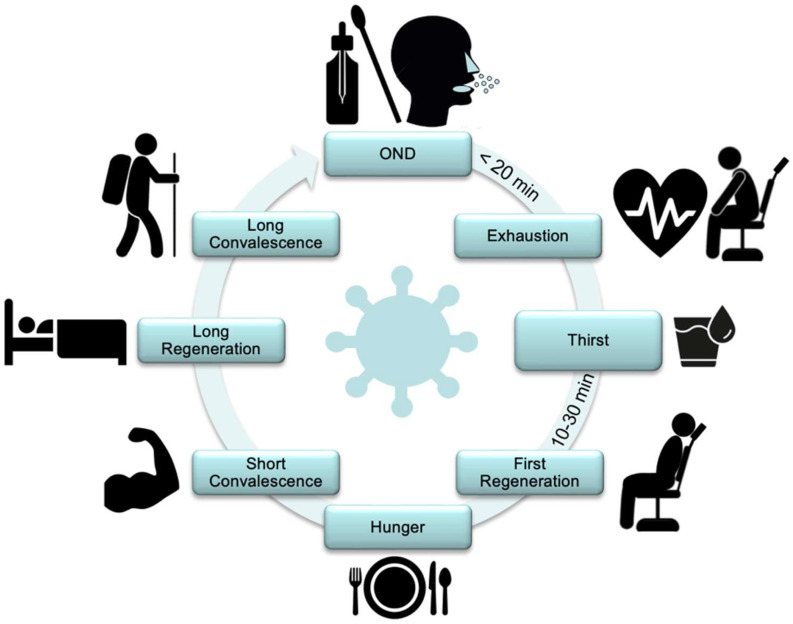
Proposed mechanism of Oronasal Drainage (OND) for Long COVID treatment. Cycle chart outlining the hypothesized treatment cycle for OND. The proposed mechanism involves OND sessions lasting less than 20 min, followed by phases of exhaustion, thirst, and initial regeneration. Subsequently, hunger and food intake lead to a brief convalescence, succeeded by extended regeneration and prolonged convalescence.

Treatment sessions, lasting 15 to 20 min and interspersed with breaks for rest and recovery, serve as a prerequisite for the inherent self-healing process [37]. Regular hydration is provided to counteract fluid loss, and the drainage frequency is adapted to the patient’s exercise capacity (Figure A1).

Altogether, OND presents an innovative therapeutic strategy for Long COVID with potential impact that seamlessly blends physiological, biochemical, and fluid mechanical elements. The main goal of OND is to target the root cause of viral persistence at the entry points in the ONM, potentially resulting in significant systematic symptom alleviation for individuals suffering from Long COVID.

## 3. Case Report

### 3.1. Anamnesis

A 57-year-old female, four-time BioNTech vaccine recipient, suffered SARS-CoV-2 in September 2022, confirmed through polymerase chain reaction (Ct = 29.0). Pre-existing conditions included arterial hypertension, hypothyroidism, and type-2 diabetes, managed with Siofor, L-thyroxine, and irbesartan. Post-infection, the patient experienced severe post-exertional malaise (PEM) and was officially diagnosed with Long COVID, fibromyalgia, restless legs syndrome, and ME/CFS. Medications included rupatadine, monteluclast, prednisolone, nattokinase (100 mg, nine months), and nicotine patches (3.5–7 mg, four months). PEM, coupled with cognitive impairment and dyspnea resulted in inability to perform patient’s managerial social work role.

### 3.2. Physical Examination

The extraoral examination identified a pale, slightly yellow, adipose face. The intraoral assessment revealed an inconspicuous dentition with conservative and prosthetic restorations. According to the patient, a tongue scraper had been used every day for months; the tongue appeared enlarged, pale, and with little coating. Further, the patient reported recurrent loss of two 10-year-old dental restorations since the SARS-CoV-2 infection, requiring repeated conservative treatment.

**Figure 2:**
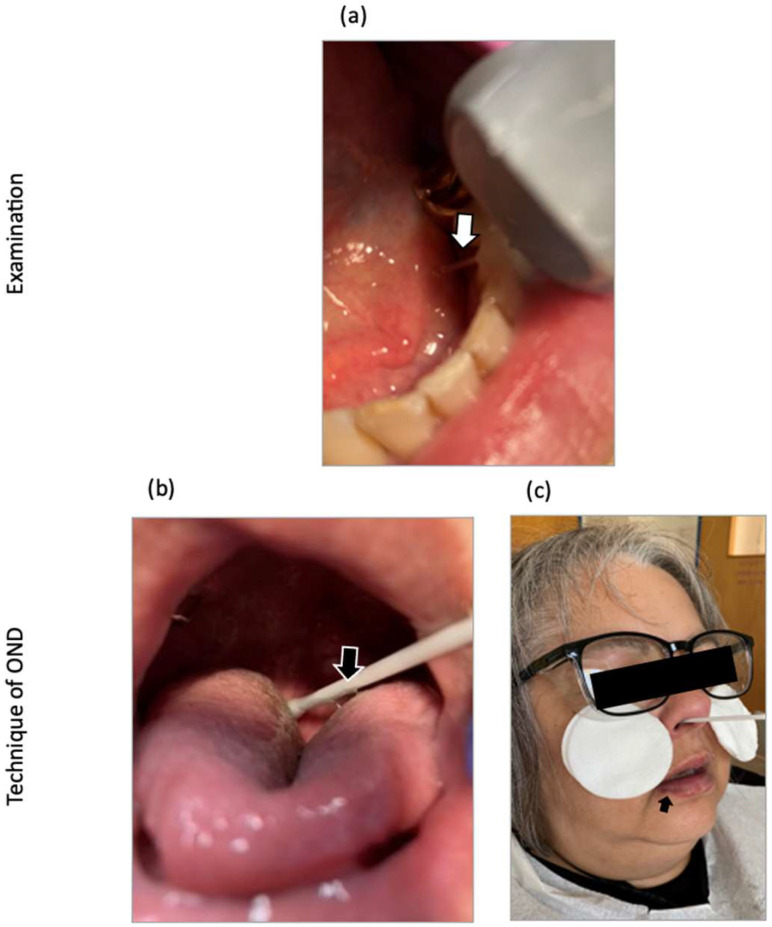
Oronasal Drainage (OND): Procedure for a Long COVID patient. (**a**) Elongated, thread-like salivary secretion observed (white arrow). (**b**) OND in the oral cavity targeting ostia on the right palatine gland. Adhesion between tongue secretions and the swab shown by the black arrow. (**c**) OND on the nasal mucosa, focusing on the epipharyngeal mucosa. Visible onset of herpes simplex virus 1 (HSV-1) reactivation on the lower lip on the fourth day of OND (black arrow).

Diagnostic imaging included intraoral and extraoral photos, along with hand images. Intraorally, the salivary secretion appeared elongated and thread-like (Figure 2a). Notably, the patient experienced significant fatigue even before the intervention while capturing the photographs.

### 3.3. Interventions

The intervention involved OND administered for five consecutive days on a dental chair, with each session comprising three to four therapy units. Due to the potential risk of vomiting resulting from a pronounced gag reflex, the patient underwent a fasting period. Initially, OND was carried out on the oral mucosa, specifically targeting the upper oral ostia next to the tonsilla palatina of the individual due to an enlarged tongue (Figure 2b). Subsequently, after a period of nutritional intake and regeneration, OND of the nasal mucosa was performed on both nostrils in the following step (Figure 2c).

### 3.4. Evaluation

Symptoms were systematically assessed before, during, and one month after the OND intervention, utilizing a comprehensive list of Long COVID associated symptoms and functional scales ranging from 0 (no symptoms) to 8 (most severe symptoms) [38]. Throughout the OND sessions, we monitored vital signs, observed changes in the HEPA device display, and captured images of the hands. Post-OND, the patient self-monitored vital signs and daily symptoms in constant communication. Additionally, a histological examination through enzyme-linked immunosorbent assay, aimed at detecting the SARS-CoV-2 spike protein (S protein) in nasal and oral secretions from the second day of treatment, was conducted based on the patient’s explicit request and at their own expense. Statistical analyses included biserial correlation and the Cochran Q test.

### 3.5. Results

Patient’s resting heart rate was 81.0 three days before the start of OND (*n* = 1), averaging 75.6 ± 4.7 during therapy (*n* = 5), and averaging 78.2 ± 3.1 at the one-month follow-up (*n* = 30). Throughout OND therapy, blood pressure ranged from an average of 129.2 ± 5.6 to 75.0 ± 2.5 (*n* = 5), and at the one-month follow-up, it ranged from an average of 123 7 ± 7.2 to 77.0 ± 5.8 (*n* = 30). Oxygen saturation averaged 93.8% ± 3.0 during OND (*n* = 5), with the lowest saturation recorded at 90% on the second day.

**Figure 3:**
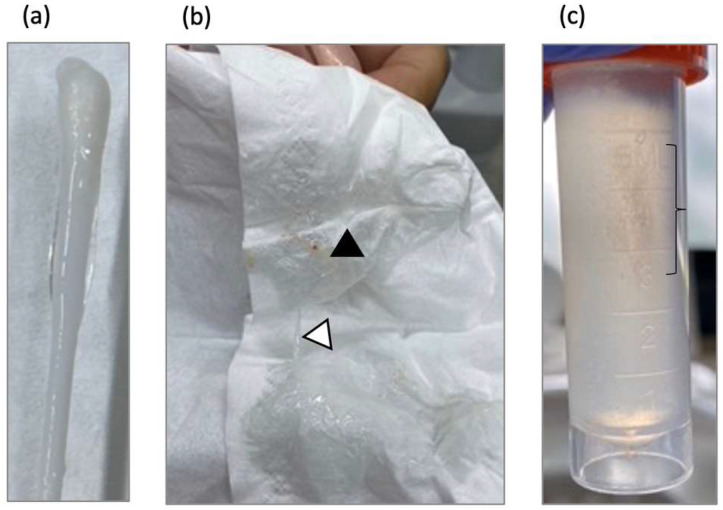
Removed nasal mucosa secretion during Oronasal Drainage (OND). (**a**) Stringy, transparent mucus measuring several centimeters. (**b**) Patient expelled long, stringy, transparent secretions through snorting (white triangle). On the fourth day of OND, a few green nasal secretions were observed (black triangle). (**c**) Nasal-mouth secretions. No S proteins were detected; the content, especially within brackets, remains unclear.

The HEPA filter was regularly very low (5–6/100), reaching higher values when sneezing related to OND (13/100) [17]. The texture of the secretion collected from both nasal and oral mucosa manifested as a stringy, transparent mucus measuring several centimeters in length (Figure 3a–c, white triangle). By the fourth day of OND, minimal quantities of green nasal secretion were expelled through snorting (Figure 3b, black triangle). With an analytical sensitivity of unbound S protein detection of 4.5 pg/mL, no S proteins were detected in the nasal-mouth secretions examined (Figure 3c).

Upon the initiation of OND, the individual encountered a flu-like experience such as watery eyes, sneezing, sniffling, coughing, a fleeting sensation of fever, back pain, and momentary exhaustion. These indications peaked on the second day of treatment and subsided on the tenth day post-treatment initiation (Figure 3b, black arrow). In addition, the patient reported a metallic taste and developed HSV-1 on the lower lip on the fourth day of OND (Figure 2c, black arrow), with both symptoms resolving by the eighth day.

**Figure 4:**
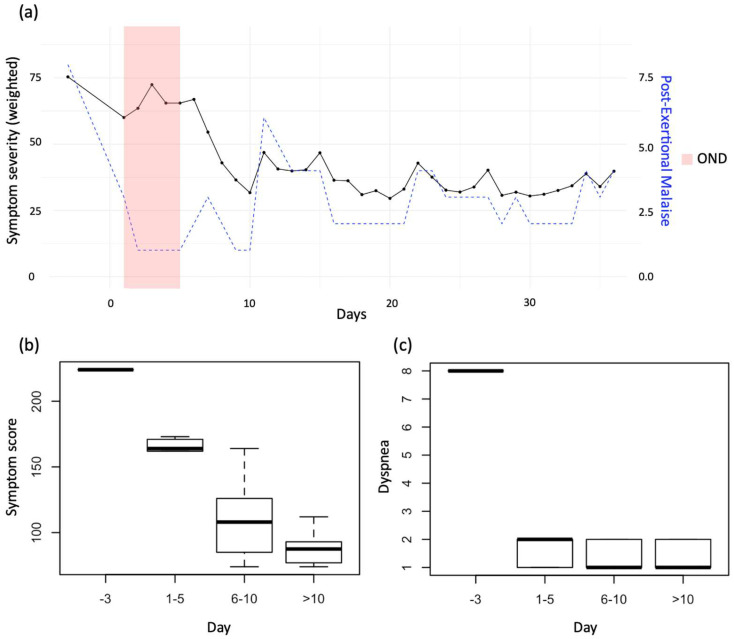
Impact of Oronasal Drainage (OND) on Long COVID Symptom Severity. (**a**) Weighted symptom severity score before, during (red bar) and after OND. Black data points represent symptoms (0 to 100, left y-axis) with mean displayed. Blue data points represent exercise intolerance, identified as post-exertional malaise (PEM) (0.0 to 1.0, right y-axis) with mean displayed. (**b**) The data encompass the complete symptom score. A thick horizontal bar within the boxplot represents the mean of symptom (0 to 250) with error bars denoting the standard error of the mean. (**c**) Symptom severity for dyspnea. A thick horizontal bar within the boxplot represents the mean of symptom (0 to 8) with error bars denoting the standard error of the mean; (**b**,**c**) reflect data collected before (−3 days), during (1–5 days), immediately after (6–10 days), and during follow-up (>10 days; *n* = 35) OND sessions.

Symptom improvements exhibited a positive correlation with OND, indicating moderate effects on the tenth day post-treatment initiation (Cohen’s d = 0.44, Figure 4a, black data). These effects persisted and remained stable one month following the conclusion of the treatment (Figure 4b).

**Figure 5:**
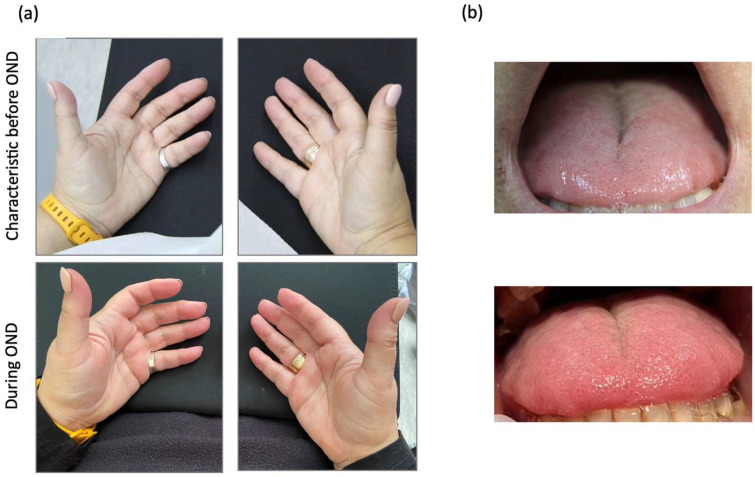
Impact of Oronasal Drainage (OND) on Long COVID skin. (**a**) The upper panel illustrates the patient’s hands before OND, while the lower panel shows the patient’s hands on the fourth day of OND. (**b**) The upper panel illustrates the patient’s tongue before OND, while the lower panel shows the patient’s tongue on the fifth day of OND.

OND treatment resulted in significant improvements in PEM (Figure 4a, blue data), dyspnea (Figure 4c), respiratory issues, abdominal pain, nausea, sensorimotor complaints and food intolerances (Table A1) [38]. Remissions were observed in reproductive organs, allergies and diarrhea (Table A1, green data). Improvements were accompanied by visibly enhanced blood circulation in the hands (Figure 5a) as well as slight enhanced blood circulation in patient’s tongue tip (Figure 5b). The absence of PEM during OND and the sustained improvement in symptoms enabled the individual to resume volunteer duties including public appearances.

## 4. Discussion

### 4.1. In General

The innovative OND resulted in moderate symptom improvements across multiple systems (systemic, pulmonary, cardiovascular, gastrointestinal, musculoskeletal, and neuropsychiatric domains) in an individual affected by Long COVID. Furthermore, resting heart rate slightly improved. Blood pressure also had a tendency to normalize.

The case report showcases notable strengths, demonstrating substantial alleviation of Long COVID manifestations in a single patient. Furthermore, the remission of gynecological and gastrointestinal complaints, along with marked relief in food intolerance, underscores the positive impact of this novel treatment approach for Long COVID. Notably, the absence of PEM during OND is noteworthy, especially considering that existing literature provides only broad self-management strategies for ME/CFS [39]. Among the most prevalent Long COVID symptoms, fatigue showed a slight improvement, while dyspnea drastically improved during OND [40]. Additionally, OND emerged as an effective, cost-efficient intervention with a relatively brief treatment duration.

This case report is subject to certain limitations. The patient could not assess oxygen saturation afterward due to a lack of equipment. Notably, the temporary marginal decrease in oxygen saturation during OND contrasts sharply with the marked improvement in dyspnea observed during and post-therapy [40]. Enhanced blood circulation in hands and tongue, indicative of systemic improvements, was not conducted for ethical reasons due to limits as a healing attempt. The use of a tongue scraper by the patient to address a coated tongue, associated with SARS-CoV-2 virus persistence, introduces a potential confounder [10]; however, its impact seems marginal considering the patient’s long-term use.

Moreover, there is currently no identified evidence indicating dental restoration loss in this population based on our knowledge, reflecting limited data. The necessity for a rigorous histological examination of nasal-mouth secretions, along with broader, randomized studies, underscores the preliminary nature of the findings. The cost-effectiveness of OND makes studies akin to this case report relatively straightforward to conduct. While the lack of blinding raises concerns about potential psychosomatic influences [41], a three-month post-intervention follow-up showed no observed symptom-related relapses, supported by age-appropriate normal findings on cranial MRI.

### 4.2. Laboratory Findings—SARS-CoV-2 S Protein

During OND, a slight increase in aerosols indicated non-specific contamination, potentially involving SARS-CoV-2 particles [17]. Existing literature supports the persistence of S proteins from SARS-CoV-2 [11]. However, the absence of histologically detectable S protein in nasal-mouth secretions may be attributed to various factors. Firstly, targeting exclusively unbound S proteins might have led to insufficient measurement sensitivity. Secondly, a histological examination for Nucleocapsid protein [11,12], Envelope protein, or RNA [15] was not carried out due to the absence of precise determination methods. Thirdly, OND intervention twelve months post-acute infection might have contributed, considering the decreasing receptor binding of S protein over the disease course [12]. Additionally, the prolonged use of nattokinase, a time-dependent protease inhibitor, and nicotine patches four months prior could influence S protein inhibition and receptor binding, respectively [42,43].

Another report demonstrated SARS-CoV-2 RNA persistence in the epipharyngeal mucosa four months post-infection, eliminated with EAT [9]. However, the higher degradation rate of viral RNA compared to proteins reduces RNA detection likelihood after twelve months in this case [11]. Further diagnostic analysis is imperative, especially focusing on the Nucleocapsid protein, which constitutes the innermost component of SARS-CoV-2 [44].

### 4.3. Side Effects

Side effects, interpreted as subacute infection signs, highlight the need for careful monitoring during OND [35]. Alterations in taste and the reactivation of HSV-1, recognized self-limiting and manageable symptoms following a SARS-CoV-2 infection [36], occurred during OND, and strengthen suspicion of infected secretions.

The green coloration in nasal secretion after four days of OND may stem from pre-existing bacterial persistence [45]. The appropriateness of antibiotics remains debatable, given the very small quantity and patient’s relative well-being despite strenuous treatment. Regarding these well-tolerated manifestations as transient side effects associated with OND, we found no urgency for further intervention.

### 4.4. Proposed Mechanisms

SARS-CoV-2 predominantly enters through the nasopharynx [46]. Post SARS-CoV-2 infection, genetic changes in monocytes [47], infection of foam cells [48] and macrophages [47] suggest a potential role of nasal-mouth secretion in the context of the MALT immune system [35]. Recognizing viral persistence [10,11,12,13,14,15], the ostia in the ONM emerge as primary sites for SARS-CoV-2 persistence. Employing a novel EAT approach [6,7,8] that demonstrates mRNA removal [9], OND holds promise as an effective treatment for virus elimination. The hypothesized actions encompass the opening of ostia, equalization of chloride ion concentration gradients using a ZnCl_2_ solution with a specific emphasis on the ORF3a protein of SARS-CoV-2 [28,44]. The high unbinding force of angiotensin-converting enzyme 2 may explain the long secretion thread during OND [49], potentially involving a chaining of infected cells as the underlying mechanism.

### 4.5. Comparison to EAT

Enhancements in both fatigue and headache are observed in EAT treatment [7], emphasizing that OND innovatively expands upon EAT with novel techniques and extended therapeutic applications. In contrast to EAT, OND avoids injuries and bleeding during the treatment process, thereby preventing pain and infection risks, and enhancing patient comfort [6]. Furthermore, a clinical study investigating oral bloody abrasion in the context of EAT, which resulted in an elevated heart rate in patients with ME/CFS, stands in contrast to the presented case [30]. Here, the patient with Long COVID and ME/CFS exhibited a slight improvement in heart rate. Moreover, OND’s comprehensive influence on the patient’s overall health stands in contrast to EAT’s specific focus on addressing chronic cough [7]. Consequently, OND is generally considered superior to EAT.

### 4.6. Future Research

In subsequent studies, to address the initial exacerbation resembling the onset of a flu-like infection, peaking after two days, OND should be monitored via clinical thermometer and temporarily suspended for one day after a two-day treatment duration. This pause is intended to facilitate the regeneration process.

The frequency of therapy sessions should vary based on viral load, disease duration, and severity, with 20–40 treatments within 24–36 weeks being common for EAT and potentially applicable for OND [6,15,16,50]. While the current case report does not include diagnostic assessments of viral load or disease duration due to ethical constraints, these aspects should be systematically investigated in future clinical studies. Moreover, given the minimal risk of PEM, the assessment of hand grip strength could serve as a straightforward diagnostic tool for evaluating systemic alterations [51].

### 4.7. Lymphatic Tissue Involvement in Long COVID: A Hypothesized Mechanism and Potential Therapeutic Approach

Following a SARS-CoV-2 infection, inadequate viral elimination may be the primary factor contributing to the onset of the secondary condition known as Long COVID [15,52]. Our hypothesis suggests Long COVID as a disorder related to the lymphatic tissue, taking into consideration viral entry points [46], replication of SARS-CoV-2 across ocular tissue, nasopharynx and oropharynx [14], as well as viral persistence in the pharyngeal lymphoid tissue [25] and the enlargement of cervical lymph nodes [53]. Furthermore, our hypothesis considers factors such as immune response attack on spleen and lymphoid nodes [54], perithymic lymphadenopathy linked to severe disease and death [55], and the removal of previously infected MALT cells [9]. Decontamination and reactivation of all infected ostia in the mouth, nose, and eyes could play a pivotal role in addressing Long COVID symptoms possibly associated with these openings [14,21,23,24]. Consequently, targeting ostia in the ONM, OND emerges as a potential key component in Long COVID treatment. However, further research is crucial to validate these hypotheses, considering factors such as SARS-CoV-2 viral load and the involvement of MALT and ostia in viral diseases [15,16,50].

## 5. Conclusions

In conclusion, OND introduces a novel and carefully considered therapeutic approach for Long COVID. By integrating physiological, biochemical, and fluid mechanical components, OND aims to address specific aspects of viral persistence within the ONM. Despite experimental stages and temporary side effects, OND emphasizes safety commitment and presents a unique avenue for investigating potential solutions to the lingering effects of Long COVID. As we cautiously navigate the landscape of post-acute sequelae, OND offers a noteworthy contribution to the ongoing discourse on effective treatments for individuals experiencing prolonged complaints after SARS-CoV-2 infection. The case report suggests that OND may lead to rapid symptom alleviation and functional recovery. However, further research and clinical trials will be essential for robust conclusions to determine the true impact and feasibility of OND in Long COVID management.

## Data Availability

The original contributions presented in this study are included in the article. Further inquiries can be directed to the corresponding author.

## Appendix A

**Table A1:** Improvements of Long COVID associated symptoms through Oronasal Drainage (OND), before, during and post treatment.

			Symptom Severity					
	*OND Treatment*	Before	While OND		Follow-Up After OND			
	*Days*	−3	1–5		6–10		10–35	
	*n*	1	5		5		25	
Category	Symptom	Mean	Mean	SD	Mean	SD	Mean	SD
systemic	fatigue	7.00	6.40	0.89	5.60	0.89	5.00	0.89
	PEM	8.00	1.40	0.89	1.80	0.83	3.04	1.11
	sweats	8.00	5.20	1.64	4.60	0.55	3.77	1.27
	temperature issues	7.00	6.80	0.84	5.00	0.00	3.85	1.19
pulmonary	awakened feeling of not being able to breathe	2.00	0.00	0.00	0.00	0.00	0.00	0.00
	dry cough	3.00	2.00	1.00	1.00	0.00	1.00	0.28
	respiratory issues	8.00	2.80	1.64	1.60	0.89	0.92	0.63
	dyspnea	8.00	1.60	0.55	1.40	0.55	1.35	0.49
cardiovascular	high blood pressure	4.00	0.60	1.34	0.00	0.00	0.15	0.46
	palpitations	4.00	1.40	0.55	1.00	0.00	1.04	0.20
	tachycardia	5.00	3.80	0.84	1.40	0.55	1.58	0.58
	tightness of chest	3.00	2.20	1.10	2.20	0.84	1.23	0.51
gastrointestinal	abdominal pain	7.00	0.80	1.30	0.60	0.55	0.81	0.90
	diarrhea	7.00	1.20	1.64	0.00	0.00	0.27	0.78
	nausea	8.00	4.60	3.78	1.80	1.30	1.46	0.71
	vomiting	4.00	2.60	3.71	0.00	0.00	0.00	0.00
musculoskeletal	joint pain	7.00	1.60	0.89	1.40	0.55	2.04	0.92
	muscle aches	7.00	1.40	0.55	1.60	0.55	2.50	1.07
neuro-psychiatric	brain fog	8.00	3.60	1.14	5.80	0.45	3.96	1.18
	change of smell and taste	4.00	5.40	1.14	2.80	1.10	1.27	0.72
	dizziness	8.00	7.80	0.45	6.60	0.89	4.15	0.37
	headache	6.00	4.20	1.10	3.40	0.55	2.50	0.91
	insomnia	3.00	2.80	1.48	1.60	1.34	2.46	1.42
	neuralgia	2.00	0.60	0.55	0.00	0.00	0.00	0.00
	other sleep issues	5.00	2.80	2.77	1.60	1.95	1.73	0.83
	restless legs	5.00	0.00	0.00	0.60	1.34	0.81	0.80
	sensorimotor issues	8.00	3.00	0.00	2.40	0.55	1.04	0.20
	sleep apnea	2.00	0.00	0.00	0.00	0.00	0.00	0.00
immunologic	food intolerances	8.00	5.40	1.67	3.00	1.00	1.73	0.72
	new allergies	8.00	0.00	0.00	0.00	0.00	0.12	0.43
endocrine, reproductive	bladder control issues	8.00	2.20	0.45	3.00	0.00	3.58	0.50
	reproductive organs	4.00	1.80	2.49	0.00	0.00	0.00	0.00
head, ear, eye, nose, throat	hearing issues	3.00	2.80	0.45	1.20	0.45	1.00	0.00
	runny nose	4.00	2.00	1.22	1.20	0.45	0.77	0.82
	sore throat	4.00	2.20	2.05	1.00	0.00	1.00	0.40
	tinnitus	2.00	0.40	0.55	0.00	0.00	0.15	0.46
	vision symptoms	5.00	5.20	0.45	1.20	0.45	2.04	0.20
oral	dental issues	3.00	0.20	0.89	0.60	0.89	0.73	0.60

The traffic lights represent the severity of Long COVID symptoms, rated on a scale from 0 (no symptoms = green) to 8 (most severe symptoms = red), observed in an individual who underwent OND. Symptom progression was tracked three days before, during the five-day treatment, and 30 days post-treatment. Enhancements between 0 and 1 point were omitted for improved readability. PEM = post-exertional malaise. SD = standard deviation.
